# Analysis of continuous biochemical parameters in long-term trials of chronic disease where outcomes could be affected by further intervention

**DOI:** 10.1186/1745-6215-16-S2-P62

**Published:** 2015-11-16

**Authors:** Natalie Ives, Samir Mehta, Elizabeth Brettell, Marie Valente, Sunil Bhandari

**Affiliations:** 1 University of Birmingham, Birmingham, UK; 2 Hull and East Yorkshire Hospitals NHS Trust, Hull, UK

## Background

Chronic kidney disease (CKD) affects 1 in 10 adults in the UK, and is an increasing burden on the NHS - dialysis costs ~£30,000/year. Key clinical outcomes in CKD are need for dialysis or transplantation and survival. However, designing trials with these endpoints requires large patient numbers. To reduce sample size requirements, trials have used measures of renal function (e.g. serum creatinine, glomerular filtration rate), which correlate with longer-term clinical outcomes. However, there are interpretation issues for these parameters if the rate of dialysis or transplant is affected by treatment group, as these interventions impact on renal function.

## Methods

We plan to undertake a review of studies in stage 4/5 renal disease to assess analysis methods used. Have trials excluded patients from the analysis when they start dialysis or have a transplant, or if they are included, what methods have been used to account for this or potential data missing not at random. Possible options include last observation carried forward, flat value or multiple imputation. Based on this review, we plan to assess the different analysis methods in terms how results may vary and which provides the most robust result.

## Results

The results will inform the statistical analysis plan for the STOP-ACEi trial (funded by NIHR EME) assessing withdrawal of ACE-inhibitors and Angiotensin Receptor Blockers in patients with advanced CKD.

## Conclusions

Although this example relates to renal disease, there are other areas where continuous outcomes are used in trials, where other factors may influence or impact on this outcome.

